# An Update in the Use of Antibodies to Treat Glioblastoma Multiforme

**DOI:** 10.1155/2013/716813

**Published:** 2013-11-05

**Authors:** Norma Y. Hernández-Pedro, Edgar Rangel-López, Gustavo Vargas Félix, Benjamín Pineda, Julio Sotelo

**Affiliations:** ^1^Neuroimmunology and Neuro-Oncology Unit, Instituto Nacional de Neurología y Neurocirugía, Mexico City 14269, Mexico; ^2^Excitatory Amino Acids, Instituto Nacional de Neurología y Neurocirugía, Mexico City 14269, Mexico

## Abstract

Glioblastoma is a deadly brain disease and modest improvement in survival has been made. At initial diagnosis, treatment consists of maximum safe surgical resection, followed by temozolomide and chemoirradiation or adjuvant temozolomide alone. However, these treatments do not improve the prognosis and survival of patients. New treatment strategies are being sought according to the biology of tumors. The epidermal growth factor receptor has been considered as the hallmark in glioma tumors; thereby, some antibodies have been designed to bind to this receptor and block the downstream signaling pathways. Also, it is known that vascularization plays an important role in supplying new vessels to the tumor; therefore, new therapy has been guided to inhibit angiogenic growth factors in order to limit tumor growth. An innovative strategy in the treatment of glial tumors is the use of toxins produced by bacteria, which may be coupled to specific carrier-ligands and used for tumoral targeting. These carrier-ligands provide tumor-selective properties by the recognition of a cell-surface receptor on the tumor cells and promote their binding of the toxin-carrier complex prior to entry into the cell. Here, we reviewed some strategies to improve the management and treatment of glioblastoma and focused on the use of antibodies.

## 1. Introduction

Since the “magic bullet” concept proposed by Paul Ehrlich more than one century ago in which he describes that specific recognition and elimination of pathogen organisms or malignant cells by antibodies (Abs) is possible, many types of these molecules have been developed as tools against cancer. Abs have the capacity to travel through the blood, binding to specific tumor antigens on the surface of cells or recognizing other “tumor-related” targets, blocking ligand-receptor growth signals, some survival pathways, and finally eliciting tumor cell death [1].

Neuroephitelial tumors are the most common primary intracranial tumors of the central nervous system (CNS), and, unfortunately, malignant gliomas are the most lethal type of adult brain tumors. According to the World Health Organization (WHO), the classification of malignant gliomas is based on morphological similarities of the tumor cells with nonneoplastic glial cells. Therefore, gliomas have been classified and graded on a malignant scale from I to IV as follows: astrocytic (grade I–IV), oligodendroglial (grade II-III), mixed oligoastrocytic (grade I–III), and ependymal tumors (grade I-II). Particularly, glioblastoma multiforme (GBM) is an anaplastic cellular, grade IV tumor with pleomorphic astrocytic cells with marked nuclear atypia and high mitotic rates [2]. Glioblastomas are rapidly evolving tumors typically with neoplastic infiltration of adjacent normal brain tissue and solid proliferating tumor at the periphery. Primary GBM arises de novo, whereas secondary GBM develops from preexisting low-grade astrocytomas [3]. Primary and secondary GBM are clinically indistinguishable. However, genotypically, there are some differences between them, which could be used in the search for improved treatment [3, 4]. Some of the genetic changes found in gliomas include amplification and/or overexpression of oncogenes, loss of tumor suppressor genes, DNA repairing genes through mutation, loss of heterozygosity (LOH) in some chromosomes, or epigenetic mechanisms such as hypermethylation of promoters. These genetic changes result progressively in uncontrolled proliferation rates and loss of normal cell cycle control mechanisms, diminishing the ability of cells to undergo apoptosis in response to genotoxic agents, failure of DNA repairing mechanisms, increasing genetic instability, and deregulation of growth factor signaling pathways [5–7].

Glioblastoma tumors are heavily infiltrated by cells of myeloid origin, mainly microglia and macrophages [8]. These glioma-infiltrating myeloid cells (GIMs) comprise up to 30% of the total tumor mass and they have been implicated in several roles during GBM progression including proliferation, survival, motility, and immunosuppression. The origin of these GIMs seems to be from both resident brain macrophages (microglia) and newly recruited monocyte-derived macrophages from the circulation [9].

**Figure 1:**
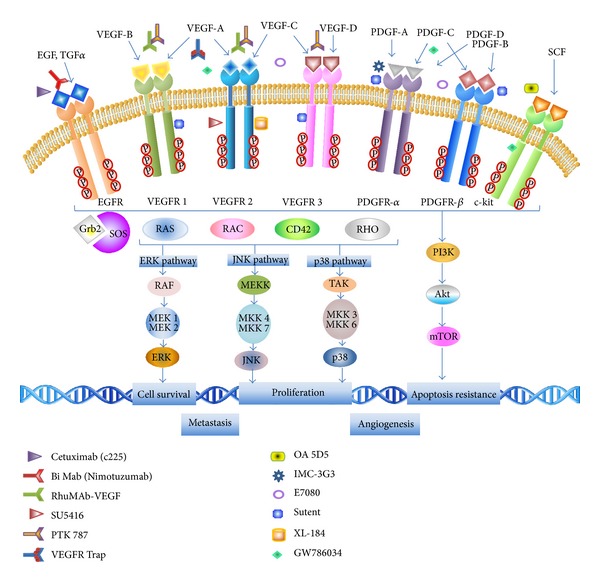
Antibodies used in gliomas treatment. Inhibition of tyrosine kinase downstream pathways signaling modulated by monoclonal antibodies to EGFR, VEGFR, PDGFR, and c-kit. Cdc42: cell division control protein 42, ERK: extracellular signal-regulated kinase, mTOR: mammalian target of rapamycin, PI3K: phosphatidylinositol 3-kinase, EGF(R): epidermal growth factor (receptor), Grb2: growth factor receptor-bound protein 2, JNK: c-Jun N-terminal kinase, MEK/MKK: mitogen-activated protein kinase kinases, PDGF(R): platelet derived growth factor (receptor), SOS: son of sevenless, TAK: TGF*β*-activated kinase, TGF: transforming growth factor, and VEGF(R): vascular endothelial growth factor (receptor). Adapted and modified from Giamas et al. [30].

Despite the use of aggressive multimodality therapies that include surgery, radiotherapy, and chemotherapy, the median survival is only from 12 to 15 months. Additionally, the standard treatments for these tumors often result in debilitating motor and neurological deficits that alter physical skills and diminishing the quality of life of these patients. Nowadays, the literature describes the development of new strategies that could increase the prognostic and diminish the adverse events in patients. The known biology of glial tumors has allowed proposing some predictive markers that could be used to try a personalized treatment against gliomas. Between these markers is notable the role played by growth factors, such as the epidermal growth factor and the vascular epidermal growth factor, in gliomas progression and its treatment (Figure 1). 

## 2. Role of Growth Factor Receptors in Tumorigenesis and Cancer Progression

The epidermal growth factor (EGF) has been implicated in supporting oncogenesis and progression of human solid tumors. EGF promotes tumor development amplifying the expression its tyrosine kinase epidermal growth factor receptor (EGFR) by increasing ligand-activated signaling through of its own receptor [31]. EGF plays a central role in cancer development since it is involved in crucial steps of tumor progression such as proliferation, angiogenesis, invasiveness, decreased apoptosis, and loss of cellular differentiation. In primary gliomas, the frequency of amplification of EGFR has been reported around in 40% of the examined cases [32].

Besides, several types of EGFR gene mutations have been reported in many tumors, including in GBM, and in nearly all cases these alterations have been related to EGFR amplification. Particularly, the mutant EGFR class III variant (so-called EGFRvIII) contains a deletion of 267 amino acids of the extracellular domain which creates a mutant with a unique extracellular domain [32]. This mutant EGFRvIII is ligand independent and it has been associated with constitutive activation of the wild receptor and failure to attenuate signaling by receptor downregulation. Also, it causes mitogenic effects, and it exhibits a more powerful transforming activity [33, 34]. In this way, the constitutively active EGFRvIII can enhance cell proliferation in part by downregulation of p27 through activation of the phosphatidylinositol 3-kinase/serine-threonine kinase alpha (PI3K/Akt) pathway [35, 36].

Recent advances in targeted therapies have demonstrated that tyrosine kinase inhibitors (TKIs) have provided a marked benefit to subsets of patients whose tumors harbor specific genetic abnormalities. However, patients with EGFR mutations rapidly acquire resistance to TKI inhibitors decreasing the median time to disease progression to a few months [37].

Several strategies had been envisioned to overcome this resistance, such as dual-target inhibitors and multitarget and combined therapies. *In vitro* and *in vivo* properties and antitumor efficacy of the anti-EGFR/anti-CD3 bispecific monoclonal antibody (biMAb), so called M26.1, have been analyzed in previous reports. Treatment of IGROVI tumor-bearing mice with activated human lymphocytes coated with M26.1 F(ab')2 significantly prolonged survival of the animals compared with tumor-bearing untreated mice. Therefore, these results strongly suggest the clinical usefulness of bispecific M26.1 F(ab')2 as a targeting agent for local treatment of tumors such as glioma and ovarian cancers that express variable levels of EGFR [38].

Nowadays, some monoclonal antibodies (mAbs) have been developed that act or bind directly to EGFR mutated. Between these molecules, mAb-806 is a monoclonal anti-EGFRvIII antibody which significantly reduced the volume of tumors and increased in 61.5% the survival of mice-bearing xenografts of EGFRvIII gliomas compared to controls [39]. Patel and co-authors report that the mAb Cetuximab (c225), successfully targets and binds to U87MG cells expressing high levels of EGFRVIII leading to the internalization of the complex Cetuximab-EGFRVIII. A subsequent reduction was observed in the phosphorylated form of the mutant receptor in transfected cells and in a remarkable reduction (40–50%) in cell proliferation [40]. Y10 is another antibody specific for EGFRVIII whose intratumoral injection improved survival in animal models [41]. A range of potential therapies that target EGFR, or its constitutively active mutant EGFRVIII, are currently in development or in clinical trials for the treatment of GBM. Data from experimental studies evaluating these therapies have been very promising; however, their efficacy in the clinic has so far been limited by both upfront and acquired drug resistance in patients with recurrent high-grade gliomas [42].

## 3. Vascular Endothelial Growth Factor

Angiogenesis is the normal process by which new vessels are formed from preexisting vasculature. It is a physiological development that occurs in wound healing and when cells are exposed to hypoxia. Angiogenesis is driven by a wide variety of proangiogenic factors, mainly vascular endothelial growth factor (VEGF), and endogenous angiogenic inhibitor [43, 44]. VEGF consists of a family of 5 glycoproteins named VEGF-A, VEGF-B, VEGF-C, VEGF-D, and placental growth factor. They bind with their corresponding tyrosine kinase receptors (VEGFR-1, VEGFR-2, and VEGFR-3), activating a downstream signal, such as (PI3K), serin/trionine protein kinase alpha (Akt), and mitogen-activated protein kinase (MAPK), eliciting the development of angiogenesis and increasing vascular permeability, and the growth of lymphatic vessels that drain extravasated fluid, proteins, and tumor cells (lymphangiogenesis) [45].

In gliomas, it has been demonstrated that angiogenesis is an essential process that supplies oxygen and nutrients to developing tumors [46, 47]. The proangiogenic factors, mainly VEGF and endothelial, stromal, and tumoral cells, led to vessel growth and tumor expansion [48–50]. On base of these characteristics, some studies have been developed, with bevacizumab being the more tested.

Bevacizumab or RhuMAb-VEGF (Genentech) is a humanized monoclonal immunoglobulin G1 (IgG1) antibody against VEGF. Bevacizumab has a molecular weight of 149 kDa, and it selectively binds to all isoforms of human VEGF, therefore neutralizing VEGF's biologic activity through steric blockage of the binding of VEGF to its receptors VEGFR-1 and VEGFR-2 on the surface of endothelial cells [51]. In Phase I studies, bevacizumab has been safely administered alone and in combination with chemotherapy [52]. Besides, bevacizumab was associated with prolonged overall survival (OS) in phase III trials of metastatic colorectal [53] and non-small-cell lung [54] cancers and with prolonged progression-free survival (PFS) in metastatic breast [55] and renal cancers compared with placebo or chemotherapy alone [56]. In patients with recurrent gliomas, the combination of bevacizumab with irinotecan (a cytotoxic prodrug which inhibits DNA replication and triggers apoptotic cell death) showed a safe toxicity, rate response over 63%, and increase of PFS until 23 weeks compared with other treatments [57]. Although bevacizumab improves survival and quality of life, an eventual tumor progression is observed. A better understanding of resistance mechanisms to VEGF inhibitors and identification of effective therapies after bevacizumab administration to avoid tumoral progression are currently a critical step for patients suffering glioblastoma. Validated biomarkers are strongly needed for predicting which patients are more likely to benefit and for monitoring response. Additionally, the Amgen Dana-Farber Cancer Institute has started a clinical trial to determine the efficacy of AMG386 plus bevacizumab in patients with recurrent glioblastoma (Clinical Trial NCT01290263). Recently, two randomized, double-blind, placebo-controlled studies designed to evaluate first-line use of bevacizumab added to the standard of care (chemoradiation (CRT) with temozolomide) in glioblastoma. The data shown did not improve the median overall survival. Additionally, patients were stratified based on MGMT promoter methylation and a 9-gene signature; however, they did not identify a group of patients who demonstrated benefit from first-line use of bevacizumab, but patients with MGMT promoter methylation and a favorable 9-gene signature showed a strong trend towards a worse outcome. Adverse events were higher for patients who received bevacizumab as first-line therapy with respect to hypertension, deep vein thrombosis/pulmonary embolism, wound issues, gastrointestinal perforations, and significant hemorrhagic events, and around 30% were discontinued after end study. The lack in response due to that the chronic use of bevacizumab changes glioblastomas from highly vascular tumors to nonvascular ones, which often do not respond to bevacizumab in most patients [58]. Although bevacizumab has shown some beneficial outcomes in a subgroup of patients, studies regarding the biology involved in the gliomagenesis and angiogenesis are necessary.

It is known that VEGF/VEGFR signaling can be inhibited at the level of the receptor or via downstream signaling pathways. Since VEGFR uses many of the same signaling pathways as epidermal growth factor receptors (EGFR), the above-mentioned mutant of this receptor EGFRvIII and the platelet-derived growth factor receptor (PDGFR), including the PI3K/Akt and Ras-MAPK pathways [59], many of their inhibitors may also target VEGFR-mediated signaling. At the receptor level, two VEGFR inhibitors, PTK787 (Novartis) and SU5416 (Semaxanib; Sugen/Pharmacia) are currently being evaluated and have been included in North American Brain Tumor Consortium- (NABTC-) sponsored clinical trials [60]. PTK787 inhibits all three VEGF receptors (VEGFR2-KDR/Flk-1; VEGFR1-FLT-1; and VEGFR3- FLT-4) and reduces the number of tumor microvessels in an animal model [61]. Currently, it is being evaluated in GBM patients in a Phase I clinical trial [62]. The inhibitor SU5416 also targets VEGFR2, and it has demonstrated impressive results in animal models of a variety of cancers including GBM [63–65].

Aflibercept (VEGF Trap) is a recombinant fusion protein of the extracellular domains of VEGF fused to the Fc portion of IgG1, which binds with high affinity to both VEGF and PlGF. Preclinical studies in glioma animal models have demonstrated the efficacy of aflibercept to simultaneously inhibit angiogenesis and tumor invasion [66]. A recent study sponsored by the North American Brain Tumor Consortium Phase II of this recombinant protein demonstrated minimal activity in recurrent GBM [67]. However, preclinical data support a potential synergistic benefit of radiation therapy combined with aflibercept, and future studies may include combinations of this agent with radiation or chemotherapy [61]. Recently, Paz and Zhu correlate changes in cytokine and angiogenic factors as potential markers of toxicity to aflibercept [68]. They found that changes in IL-13 from baseline to 24 hrs predicted toxicities and increases in IL-1b, IL-6, and IL-10 at 24 hrs which were significantly associated with fatigue. The progression-free survival was 14.9 months for patients in the all-toxicity group and 9.0 months for patients in the on-target toxicity group compared to 4.3 months for those who did not develop any grade of toxicity. Authors conclude that profiling of IL-13 as a surrogate for endothelial dysfunction could individualize patients at risk during anti-angiogenic therapy, and identify those at higher risk for fatigue using IL-6 and IL-10 as markers [68].

Other mAb targeting the VEGFR-2 is Ramucirumab, which is a fully human monoclonal currently under development. Ramucirumab blocks VEGF binding and thwarting the angiogenic process. It is thought that inhibiting VEGFR-2 might yield superior outcomes in several solid tumors. Ramucirumab has demonstrated activity *in vitro* and in murine models against leukemia and ovarian cancer cell lines and in Phase I and II clinical trials against breast and gastric cancers [69].

Ramucirumab inhibits VEGFR-2 expression from normal endothelial cells, as well as tumor endothelial cells, impairing endothelial healing and hypercoagulability. Preliminary data suggest that ramucirumab is well tolerated, with manageable adverse effects. The safety of ramucirumab has not been reported on extensively; therefore, results from the many ongoing studies should shed light on this important area. VEGF inhibition increases the risk of bleeding events, as seen with bevacizumab (Avastin), another mAb that inhibits VEGF expression. Hypertension and renal toxicities are also not unexpected with ramucirumab. Based on safety data from trials of bevacizumab, investigators decided to exclude certain patient populations from subsequent trials of ramucirumab. These include patients who have brain metastases and a recent history of thrombotic events, nonhealing wounds/ulcers, and major blood vessel encasement or invasion [7].

Currently, there is an interventional open-label study sponsored by the National Cancer Institute and ImClone LLC, where investigators plan to enroll 80 patients with brain and central nervous system tumors, particularly recurrent glioblastoma multiforme. One group will receive ramucirumab intravenously administered; the other group will receive anti-PDGFR*α* monoclonal antibody IMC-3G3. Both treatments will be continued until disease progression or unacceptable toxicity. The primary outcome measure for this trial is progression-free survival at 6 months. Secondary outcome measures include objective tumor response rate, overall survival, acute and late toxicities, pharmacokinetic and pharmacodynamic profiles, and immunogenicity (Clinical Trials.gov Identifier number NCT00895180).

Vandetanib (ZD6474) is an oral inhibitor that targets VEGFR, RET tyrosine kinase receptor family inhibitor, and the EGF receptor [70]. Treatment with vandetanib in a BT4C rat glioma model significantly altered the protein expression pattern in malignant glioma and normal brain [71, 72]. Following completion of a Phase I study of vandetanib, radiotherapy, and temozolomide in patients with newly diagnosed GBM, it was concluded that this inhibitor can be safely combined with radiotherapy. A Phase II study in which patients were randomized to receive vandetanib (100 mg) daily with radiotherapy and temozolomide or radiotherapy and temozolomide alone is currently underway [71].

Vatalanib (PTK787, ZK222584, or PTK/ZK) is an orally active, small-molecule VEGF R-TKi that inhibits all known VEGFRs, as well as PDGFR-*β* and c-KIT, but is most selective for VEGFR-2. A Phase I pharmacokinetic study of vatalanib plus imatinib (a tyrosine kinase inhibitor which prevents phosphorylation and the subsequent activation of growth receptors) and hydroxyurea in recurrent malignant glioma patients determined that vatalanib at doses of up to 1000 mg twice a day combined with imatinib and hydroxyurea was well tolerated and may enhance antiangiogenesis activity [3]. Similar tolerance of this agent was found in a Phase I trial with biomarker studies of vatalanib in patients with newly diagnosed GBM treated with enzyme-inducing antiepileptic drugs and standard radiation and temozolomide [4]. An EORTC Phase I/II study on concomitant and adjuvant temozolomide and radiotherapy with vatalanib in newly diagnosed GBM reported that once-daily administration of up to 1000 mg of vatalanib in conjunction with concomitant temozolomide and radiotherapy was feasible and safe. However, a planned randomized Phase II trial was aborted owing to industry decision to halt further development of this agent [73].

As previously assessed, VEGFR and EGFR play a significant role in glioblastoma angiogenesis and proliferation, making tyrosine kinase (TK) receptors logical targets for treatment. Particularly, AE788 is a novel reversible TK inhibitor of the EGF and VEGF receptors [8, 9, 46]. Recently, Reardon et al. evaluated the role of this TK inhibitor in sixty-four recurrent glioblastoma patients. Patients in group A experienced DLTs (proteinuria and stomatitis) at 550 mg; thereby 550 mg of AE788 was the highest dose evaluated and dose limiting. Patients in group B received 800 mg of AE788 and experienced diarrhea. The initially recommended dose for dose-expansion phase for Group A was 400 mg; additional patients received 250 mg to assess the hepatotoxicity. Most frequently reported adverse events (AEs) included diarrhea and rash. Serious AEs, most commonly grade 3/4 liver function test elevations, were responsible for treatment discontinuation in 17% of patients. AEE788 concentrations were reduced by EIACD. The best overall response was stable disease (17%). Continuous, once-daily AEE788 was associated with unacceptable toxicity and minimal activity for the treatment of recurrent glioblastoma. The Phase I/II study of AEE788 in patients with recurrent/relapse glioblastoma was, therefore, discontinued prematurely [47].

Cediranib is an orally available pan-VEGFR tyrosine kinase inhibitor with a half-life of 22 hours compatible with once daily dosing [44] which has a subnanomolar 50% inhibitory concentration for VEGF receptors with additional activity against platelet-derived growth factor *β* and c-Kit. In a preliminary study on a subset of patients with recurrent glioblastoma, it was observed that cediranib treatment normalizes tumor vasculature and alleviates edema [74]. Recently, the final clinical efficacy, toxicity, and biomarker data on the entire cohort of patients treated on the first Phase II study of Cediranib in GBM was investigated, and authors report that Cediranib monotherapy for recurrent glioblastoma is associated with encouraging proportions of radiographic response, 6-month progression-free survival, and a steroid-sparing effect with manageable toxicity. They identified early changes in circulating molecules as potential biomarkers of response to cediranib [75]. The efficacy of this tyrosine kinase inhibitor in combination with lomustine chemotherapy in recurrent glioblastoma is now under clinical trials Phase III to compare the use of lomustine with cediranib, cediranib alone, or lomustine with placebo to see whether the combination or cediranib alone will be more effective than the chemotherapy alone (lomustine) in preventing the growth of cancer cells.

In additional to those mentioned inhibitors, pazopanib (GW786034) is another oral agent that inhibits the tyrosine kinases associated with the VEGF, PDGF, and KIT receptors. A Phase II study has evaluated the efficacy and safety of pazopanib in recurrent GBM patients at first or second relapse and no prior anti-VEGF/VEGFR therapy. Pazopanib was administered at a dose of 800 mg daily on 4-week cycles without planned interruptions. Pazopanib was reasonably well tolerated with manageable toxicities similar to other anti-VEGF/VEGFR agents. However, efficacy was absent without meaningful prolongation of PFS. The median PFS was 12 weeks (95% CI: 8–14 weeks), and only one patient had a PFS greater than or equal to 6 months. Thirty patients (86%) had died, and median survival was 35 weeks (95% CI: 24–47 weeks). However, *in situ *biological activity was suggested by the observation of radiographic responses in some patients [49]. 

It has been reported that increased mitogenic signaling and angiogenesis, frequently facilitated by somatic activation of EGF receptor (EGFR; ErbB1) and/or loss of PTEN, and VEGF overexpression, respectively, drive malignant glioma growth. Recently, it was suggested that patients with recurrent glioblastoma would exhibit differential antitumor benefit based on tumor PTEN/EGFRvIII status when treated with the antiangiogenic agent pazopanib and the ErbB inhibitor lapatinib. It was found that the six-month progression-free survival (PFS) rates in Phase II patients (*n* = 41) were 0% and 15% in the PTEN/EGFRvIII-positive and PTEN/EGFRvIII-negative cohorts, respectively, leading to early finish of the trial. Two patients (5%) had a partial response and 14 patients (34%) had stable disease lasting 8 or more weeks. In Phase I (*n* = 34), the maximum tolerated regimen was not reached. On the basis of pharmacokinetic and safety review, a regimen of pazopanib (600 mg) plus lapatinib (1,000 mg), each twice daily, was considered safe. Concomitant EIACs reduced exposure to pazopanib and lapatinib. However, the antitumor activity of this combination at Phase II dose tested was limited. Pharmacokinetic data indicated that exposure to lapatinib was subtherapeutic in Phase II evaluation. Evaluation of intratumoral drug delivery and activity may be essential for hypothesis-testing trials with targeted agents in malignant gliomas [70]. Particularly, on 2007 was initiated a Phase II trial sponsored by the National Cancer Institute (USA) to determine the side effects and how well pazopanib works in treating patients with recurrent glioblastoma which has been completed the last February in 2013.

XL-184 (BMS-907351) is another pan-tyrosine kinase inhibitor, currently under development by Exelixis Inc. and Bristol-Myers Squibb Co., for the potential oral treatment of medullary thyroid cancer, glioblastoma multiforme, and non-small-cell lung cancer (NSCLC). The principal targets of XL-184 are the receptors to tyrosine kinase MET, RET, and VEGFR-2, but also it is reported that this drug displays its inhibitory activity against KIT, FLT3, and TEK. Preclinical studies demonstrated that XL-184 potently inhibited multiple receptor tyrosine kinases in several cancer cell lines and in animal xenograft models and that the drug exhibited significant oral bioavailability and blood-brain barrier penetration. A phase I clinical trial in patients with advanced solid malignancies indicated that XL-184 was accumulated dose-dependent way in the plasma, and it had a long terminal half-life. A Phase II trial in patients with progressive or recurrent glioblastoma (clinical trial number NCT00704288) revealed modest but promising median progression-free survival. Toxicity and side effects for the drug have generally been of low-to-moderate severity [35].

Another small-molecule tyrosine kinase inhibitor is Sunitinib malate (Sutent, SU11248), an orally active inhibitor that targets several receptors including c-KIT, VEGFR-1–3, PDGFR-*α*, PDGFR-*β*, the class III receptor tyrosine kinase Flt3, colony stimulating factor-1R, and RET. A Phase I study of sunitinib and irinotecan for patients with recurrent malignant glioma demonstrated that the maximum tolerated dose of sunitinib was 50 mg administered once a day for 4 consecutive weeks followed by a 2-week rest combined with irinotecan (75 mg/m^2^) administered intravenously for an additional week. Reported dose-limiting toxicities were primarily hematological, and nonhematological toxicities included mucositis and dehydration. However, the PFS at 6 months was 24% and only one patient out of 25 achieved a radiographic response. Further development of a regimen using the dosing schedules for the combination of sunitinib and irinotecan was subsequently suspended owing to lack of efficacy [76, 77].

Another kinase inhibitor is E7080, whose targets include VEGFR, fibroblast growth factor receptor (FGFR), and PDGFR [68]. It has been shown that E7080 inhibits tumor angiogenesis by targeting endothelial cells. A number of the targets of E7080 are also expressed on tumor cells showing direct effects on tumor cell behavior [78]. Using a panel of human tumor cell lines, the effect of E7080 on cell proliferation, migration, and invasion was determined, measuring the inhibition of FGFR and PDGFR signaling in the cells. Authors found that E7080 had little effect on tumor cell proliferation. However, it blocked migration and invasion at concentrations that inhibited FGFR and PDGFR signaling. Knockdown of PDGFR-b in U2OS osteosarcoma cells also inhibited cell migration, which could not be further inhibited in the presence of E7080. Furthermore, E7080 could not inhibit the migration of a PDGFR negative cell line. Therefore, E7080 does not significantly affect tumor cell proliferation, but it can inhibit their migration and invasion at concentrations that both inhibit its known targets and are achievable clinically. An interventional, multicenter, Phase II study is now under development in subjects with recurrent malignant glioma [52].

On the other hand, a large body of evidence suggests that the platelet-derived growth factor (PDGF) family and associated receptors are potential targets in oncology therapeutic development because of their critical roles in the proliferation and survival of some cancers and in the regulation and growth of the tumor stroma and blood vessels. Several small molecules that nonspecifically target the PDGF signaling axis are in current use or development as anticancer therapies [51, 66, 79]. However, for the majority of these agents, PDGF and its receptors are neither the primary targets nor the principal mediators of anticancer activity. IMC-3G3, a fully human monoclonal antibody of the immunoglobulin G subclass 1, specifically binds to the human PDGF receptor *α* (PDGFR*α*) with high affinity and blocks PDGF ligand binding and PDGFR*α* activation. The results of preclinical studies and the frequent expression of PDGFR*α* in many types of cancer and in cancer-associated stroma support a rationale for the clinical development of IMC-3G3 [67]. Currently, IMC-3G3 is being evaluated in Phase II clinical trials for patients with several types of solid malignancies, particularly glioblastoma multiforme, in order to determine how well IMC-3G3 monoclonal antibody woks in GBM patients [61].

Sorafenib (Nexavar, BAY 43-9006) is a multitargeted small molecule that inhibits VEGFR-2, Flt3, PDGF receptor (PDGFR), FGF receptor-1, RAF, and c-KIT. It has been tested that sorafenib exerts antiglioma activity *in vitro* and *in vivo*. The treatment of established or patient-derived GBM cells with low concentrations of this inhibitor has been shown to cause a dose-dependent inhibition of proliferation, induction of apoptosis, and autophagy. Systemic delivery was well tolerated with intracranial glioma growth being suppressed via inhibition of cell proliferation and induction of apoptosis and autophagy, thus causing reduction of angiogenesis [57]. The inhibition of signal transducer and activator of transcription 3 (STAT3) by sorafenib has also been found to contribute to growth arrest and induction of apoptosis in GBM cells [80]. The efficacy of sorafenib with standard radiotherapy and temozolomide in the first-line treatment of patients with GBM was tested in patients with newly diagnosed GBM who received concurrent radiotherapy (2.0 Gy per day; total dose 60 Gy) and temozolomide (at a dose of 75 mg/m^2^ orally on days 1–5 every 28 days) and sorafenib (at a dose of 400 mg orally twice daily). The median PFS for the entire group was 6 months (95% CI: 3.7–7 months), with a 1-year PFS rate of 16%. The median OS was 12 months (95% CI: 7.2–16 months). The outcome of this trial yielded survival data similar to what has been reported with radiotherapy and temozolomide alone, suggesting that sorafenib has minimal activity against GBM when it is incorporated into initial management [81].

## 4. Hepatocyte Growth Factor

The multifunctional growth factor scatter factor/hepatocyte growth factor (SF/HGF) and its receptor, c-Met, are important mediators of brain tumor growth and angiogenesis [82–84]. Until now, the well-known biological consequences of c-Met activation are invasion, cellular morphogenesis, motility, metastasis, immortalization, and angiogenesis. The effect achieved by tyrosine kinase inhibitors of multiple factors and pathways involved in tumor angiogenesis has demonstrated clinical benefit in some neoplasms, including glial tumors. The overexpressions of HGF and c-Met in a very high percentage of patients with solid tumors are associated with a poor outcome and could benefit from Met-targeted therapies. The response to hypoxia increases HGF release and c-Met signaling, and also enhances metastasis in untreated tumors; besides it might play an important role in the resistance to VEGF-targeted agents in cancer therapy [85]. The c-met receptor tyrosine kinase is encoded by the c-met protooncogene, and it has been widely implicated in tumor progression and invasion [86]. Both SF/HGF and c-Met are overexpressed in human glioblastomas, and these expression levels correlate with glioma malignancy grade and vascularity [87–90]. Even when overexpression of SF/HGF and/or c-Met promotes glioma growth and angiogenesis *in vivo* [91], targeting of SF/HGF with single monoclonal antibodies was found to be ineffective, and they were only effective when three antibodies were combined, suggesting that single antibodies against SF/HGF could not fully block the SF/HGF:c-Met binding [92]. Recently, a one-armed (OA) variant of the anti-c-Met antibody 5D5 [93] was developed at Genentech, which acts as a pure antagonist and it can inhibit the growth of cells dependent on SF/HGF:c-Met autocrine and paracrine signaling. Martens and coauthors developed a monovalent OA-5D5 antibody which successfully inhibited glioma growth in an orthotopic *in vivo* model [94].

## 5. Cytotoxic Antibodies Drugs against Cancer Cells

Immunotoxins are a class of antineoplastic agents comprising a modified toxin linked to a cell-selective agent, such as a growth factor or antibody, for specifically targeting cancer cells [95]. The toxin may be any poison produced by an organism, including the bacterial toxins that cause tetanus, diphtheria, and so forth, or plants and animal toxins, such as ricin and snake venom [96]. A variety of toxins, mainly from plants, fungi, or bacteria, have been characterized, structurally optimized for *in vitro* stability, activity, and safety, and evaluated in animal studies and clinical trials. These toxins generally consist of several domains: the cell-binding or cell-recognition domain, the translocation domain, which enables the release of the toxin into the cytosol, and the activity domain responsible for cytotoxicity. During the development of immunotoxins, the binding domain of these toxins is replaced by cancer-cell-specific ligands, which lead the modified toxins directly to their internalization via receptor-mediated endocytosis. Upon internalization, the catalytic domain of the toxin is cleaved in the late endosome, and it is translocated to the cytosol leading to cell death by various mechanisms [97]. 

The development of an immunotoxin involves the chemical coupling or genetic fusion of a cell-selective ligand with a complete toxin or a modified form of the toxin. Since most cytotoxic drugs have a low molecular weight (<1000 g/mol), they rapidly diffuse into tumor cells and healthy tissue. This leads to the known adverse effects, which appear either rapidly or emerge later as delayed toxicity. These unwanted side effects limit the use of potent drugs even if they achieve objective responses and seem to be beneficial for the patient. In an attempt to improve the efficacy of cytotoxic agents without raising the burden of side effects, researchers have devised strategies to prevent easy diffusion by binding the toxic drugs to macromolecules, such as antibodies, serum proteins, lectins, peptides, growth factors, and synthetic polymers [98] (Table 1).

Recombinant DNA techniques have been applied in the production of the last generation of immunotoxins to promote tumor specificity delivery, penetration, and to reduce the cost and complexity of production. The cell-binding domain of the toxin is genetically removed, and the modified toxin is fused with a ligand or with DNA elements encoding the Fv portion of an antibody in these constructs [99, 100]. The light- and heavy-chain variable fragments are either genetically linked (scFv) or held together by a disulfide bond (dsFv) [101].

Diphtheria toxin (DT) has a cell-binding domain at the C terminus (amino acids 482–539) and the A chain with ADP-ribosylation activity at the N terminus. The A chain catalyzes the transfer of adenosine diphosphate-(ADP-) ribose to EF-2, preventing the translocation of peptidyl-t-RNA on ribosomes, thereby blocking the protein synthesis and subsequently killing the cell [102–104]. A natural ligand for DT on the cell membrane is the heparin-binding epidermal growth factor-(EGF-) like precursor [105]. Recombinant DT is made by replacing the C terminal cell-binding domain with a ligand that binds to a growth factor receptor or the Fv fragment of an antibody. Variable truncation of the binding segments resulting in 389 and 486 amino acid length toxin conjugates has resulted in the formation of toxins DAB389 and DAB486, respectively [106]. Another modification of DT involves substitution of two amino acids in the B chain resulting in a new molecule cross-reacting material-107 (CRM-107) [107]. This modification reduces the nonspecific binding of DT to human cells by 8000 fold, thus increasing the toxin's tumor specificity to 10,000 fold. Unfortunately, a Phase III trial comparing Tf-CRM107 with the current gold standard treatment determined that it was ineffective, and further development was terminated [108].

*Pseudomonas aeruginosa* exotoxin A is a single peptide with three functional domains: domain Ia is the N terminal and cell-binding domain; domain II has the translocation activity; and domain III is the C terminal and it catalyzes the adenosine diphosphate (ADP) ribosylation that inactivates EF-2, which further blocks protein synthesis and causes cell death. The genetic excision of domain I results in a molecule termed PE 40 which retains its translocation function and EF-2 inhibition properties but is unable to kill human cells [109, 110]. Furthermore, removal of the domain should in turn decrease the hepatotoxicity of PE immunotoxins that is due to residual binding of domain to the hepatocyte. A genetically engineered PE molecule (so-called PE38KDEL) has amino acids 253–364 linked to amino acids 381–608 with a change in the carboxyl end of PE (KDEL) to increase cytotoxic activity [111, 112]. PE38KDEL has been fused with a targeting moiety such as the antibody Fv portion, a growth factor, or cytokine. It was observed a much higher affinity for binding to cancer cell lines than the native PE immunotoxin, and it was very toxic to malignant cells [113, 114]. A Phase I trial of an immunotoxin made with an antibody attached to domains II and III of *Pseudomonas* exotoxin and EGFRvIII resulted in the formation of a new, tumor-specific extracellular sequence. Mice were immunized with a synthetic peptide corresponding to this sequence, and positive EGFRvIII cells were purified. After, they developed an immunotoxin by fusing the scFv sequences coding for domains II and III of *Pseudomonas* exotoxin A. The immunotoxin was very cytotoxic to cells expressing EGFRvIII. The combination of high affinity, cytotoxic activity, and stability makes this immunotoxin a strong candidate for further preclinical evaluation [115] (Table 2).

Ricin-based immunotoxins are probably some of the most frequently studied immunotoxins to date. Clinical trials started as early as 1994, where ricin A chain conjugates as well as galactose binding site were used, blocked intact ricin conjugates, primarily focusing on hematological malignancies [116–118]. In metastatic brain tumors, an early clinical trial using a human TfR MAb conjugated to ricin A chain (454A12-rRA) was started administering this ricin A conjugated intrathecally to patients with carcinomatous meningitis with doses ranging from 1.2 to 1200 *μ*g [119, 120]. A cerebrospinal fluid (CSF) inflammatory response manifesting with headache, vomiting, and mental status change, occurred at doses ≥120 *μ*g. Four of the eight patients demonstrated a greater than 95% transient reduction in tumor cell counts in their CSF. One patient improved clinically, but none of the patients survived in the long term. In order to avoid the immunogenicity associated with bacterial or plant toxins, human cytotoxic proteins such as ribonuclease or granzyme B have been used to target endothelial cells in tumors or tumor cells [121]. Furthermore, the expression of cancer-related proteases provides the opportunity to convert toxins into precursor toxins by replacing the furin cleavage site with a protease expressed in cancer cells. For example, the toxin is not active until it is cleaved by furin, and the furin site can be replaced by a site cleaved by urokinase using genetic mutation [122]. Several single-chain ribosome-inactivating proteins have also been used to make targeted toxins.

However, it is difficult to obtain adequate quantities of tumor-specific T cells, and the isolation and *ex vivo* clonal expansion of cytotoxic T lymphocytes (CTLs) from patients are a long and cumbersome process. As a result, a wide and general application of this approach has been limited. Many of the limitations associated with cellular immunotherapy can be circumvented by arming polyclonal CTL with tumor-specific chimeric T-cell receptors (TCR), the so-called “T-body” approach [15]. Chimeric TCR typically consist of a tumor-antigen-specific recognition scFv element derived from a mAb and components of TCR that mediate signal transduction in the CTL [16]. The T-body has the potential to recognize specific antigens in a major histocompatibility complex-(MHC-) independent manner; the applicability of this approach has been demonstrated both *in vitro* and *in vivo*.

In other studies have been used toxins that could regulate the immune system; however, a major problem with targeted toxins is the immunogenicity caused by the toxin. Pertussis toxin (PTx), a well-known toxin isolated from *Bordetella pertussis*, exerts great activity modulating the immune system. Currently, several studies regarding the effects of PTx in cancer have been initiated. Recently, we developed a study where the pleiotropic effect of PTx in an experimental model of glioblastoma C6 was analyzed. We observed a significant decrease in tumor volume in the PTx group; this was associated with a decreased in the number of regulatory T cells (Treg) and an increase of apoptotic cells. The production of proinflammatory cytokines was increased in mRNA for IL-6; a small increase in the mRNA expression of perforin and granzyme was observed in tumors from rats treated with PTx as well. Even though this was the first study where PTx was used as adjuvant in the treatment of cancer, the toxin could have applications in the integral therapy against glial tumors [123].

## 6. Perspectives and Conclusion

The treatment of gliomas remains as a great challenge in the clinical response, free survival in patients, and inhibition of tumoral progression. Conventional methods for the treatment of brain tumors usually involve delivery of drugs via systemic circulation. High systemic drug levels are often required to achieve adequate drug concentrations at the site of the brain tumor, which usually requires increasing the dose, frequency, or duration of drug administration with the consequent systemic toxicity. The resistance to several treatments, toxicity, and early progression to malignity has leading investigational studies for the development of specific antibodies to target tumoral cells and inhibit their growth. Another important failure in cancer therapy is due to sustained antitumor effects in the tumor microenvironment long enough to achieve clinically relevant therapeutic efficacy. At present, antiglioma targeting therapy focuses on delivering specific drugs that inhibit the tumoral growth and elicit its deletion by immune system.

On the other hand, it is necessary to develop strategies that increase the ability of therapeutic antibodies to cross the brain blood barrier (BBB). The design of nanoparticles conjugates with antineoplastic antibodies offers high specificity, increasing the focal levels of drugs and eliciting the delivery of them into the tumor, which could decrease the adverse events produced by conventional systemic administration. Recently, a new approach in anticancer therapy is to conjugate drugs, such as cisplatin, into liposomes or nanoparticles that guarantee its free access through BBB eliciting high levels and permanence of drugs in tumoral sites. Moreover, decreasing the size of therapeutic antibodies to conjugate them to nanoparticles is a new approach to elicit their delivering into poorly accessible CNS tumors.

Another challenge in delivery techniques for the treatment of gliomas is the distribution of therapeutic antibodies into the solid tumors due to the differences encountered between the inner and outer levels of growth factors secreted by the tumor mass, causing the tumoral cells to have a particular response to the administered treatment depending on their location. Also, it has been observed that the hypoxia levels are different in the central part than in the periphery of tumor; therefore, this hypoxia level mediates resistance to antiangiogenic therapy [25]. The bifunctional antibodies could be able to diffuse into the overall mass, diminishing the hypoxia levels by devascularization of tumor or by the use of antiangiogenic antibodies and by inducing an immune response to specific antineoplastic toxins.

## Figures and Tables

**Table 1:** Classification of clinically used toxins based on their mechanism of action.

Classification of toxins					
Toxins	Source	Mechanism	Structure	Modifications	References
ADP ribosylating toxins					
Diphtheria toxin	*Corynebacterium diphtheria *	ADP ribosylation of EF2	Activity (A chain), translocation (T), and binding (B) domains	(a) DT486 (b) DT388 or DT389 (deletion of cell-binding domain) (c) CRM107 point (mutation in cell-binding domain of DT)	[10–13]
*Pseudomonas *exotoxin	*Pseudomonas aeroginosa *	ADP ribosylation of EF2	Binding (Ia), translocation (II and Ib), and activity domains (III)	(a) PE40 and PE40KDEL (b) PE38 and PE38KDEL (c) PE38QQR (d) PE35	[10, 13–15]

Pore-forming toxins					
Cholera toxin	*Vibrio cholera *	ADP ribosylation of Gs, a subunit of G protein	Activity (A chain) and cell-binding domains (pentameric B chain)	CET40 (domains II and III)	[13, 16, 17]

Ribosome inactivating toxins					
Holotoxins-ricin	*Ricinus communis *	N-glycosylation of 28S rRNA	Activity and binding domains	(a) Ricin (b) Ricin A chain (RTA) (c) bR (blocked ricin) (d) dgA (deglycosylated ricin A chain)	[13, 18]
Hemitoxins-saporin (SAP), pokeweed antiviral protein (PAP)	*Saponaria officinalis, Phytolacca * *americana *	N-glycosylation of 28S rRNA	Single-chain proteins without binding domain		[13, 19]

Ribonucleases					
Fungal toxins-a-sarcin, restrictocin HPR, ECP, EDN	*Aspergillus *sp*. * Human	Cleavage of 28S rRNA Degradation of RNA	Single-chain proteins without binding domain Single-chain proteins		[13, 19] [13, 20]

Some immunotoxins are presented which have been used as toxin-based therapeutic approaches in the treatment of several malignancies acting on different intracellular targets. ADP: adenosine diphosphate; EF2: elongation factor 2 during protein synthesis on the ribosome; DT: diphtheria toxin; DT388 or DT389: truncated forms of DT without the receptor-binding activity; CRM107: cross-reacting material-mutant of DT without the receptor binding; PE: *Pseudomonas* exotoxin A; PE40 and PE38: truncated forms of PE without the receptor-binding domain Ia; CET40: cholera exotoxin A; RTA: ricin toxin A; HPR: human pancreatic ribonuclease A; ECP: eosinophilic cationic protein; EDN: eosinophil-derived neurotoxin.

**Table 2:** Immunotoxins against gliomas.

Immunotoxin	Toxin used	Target antigen	Administrative route	Clinical trial phase	Number and type of tumor	Outcome	Adverse effect	References
IL-4(38-37)- PE38KDEL	(38-37) PE38KDEL	IL-4R	Intratumoral (CED)	I/II	31 (25 GBM and 6 AA)	Median survival 8.2 months; six-month survival was 52%	Headache, seizure, weakness, dysphasia, and hydrocephalus	[21–23]

IL13-PE38QQR	PE38QQR	IL-13R	Intratumoral (CED)	I/II/III	Phase II, 51 (46 GBM, 3 AA, other 2); Phase III, 296 recurrent GBM	Infusion MTIC was 0.5 *μ*g/mL; up to 6 d well tolerated; median survival 42.7 weeks (95% CI, 35.6–55.6) for GBM in Phase II and 36.4 weeks in Phase III, comparable to Gliadel Wafer	Headache, dysphasia, seizure, weakness, and pulmonary embolism	[24–26]

TP-38	PE-38	TGF-*α*	Intratumoral (CED)	I	20 (17 GBM, other 3)	Median survival 28 weeks (95% CI, 4.1–45.1)	Hemiparesis, fatigue, headache, and dysphasia	[27, 28]

Tf-CRM107	DT-CRM107	Tf	Intratumoral (CED)	I/II	44 (GBM, AA)	Median survival 37 weeks, (95% CI, 26–49); 5/34 CR, 7/34 PR, response rate 35% (95% CI, 20–54; *P* < 0.0001)	Seizure, cerebral edema	[29]

GBM: glioblastoma multiforme; AA: anaplastic astrocytoma; TGF: transforming growth factor; CED: convection-enhanced delivery; MTIC: maximum-tolerated infusate concentration; CI: confidence interval; Tf: transferrin; CR: complete response; PR: partial responders; RR: radiographic response.
